# Additive Effect of Qidan Dihuang Grain, a Traditional Chinese Medicine, and Angiotensin Receptor Blockers on Albuminuria Levels in Patients with Diabetic Nephropathy: A Randomized, Parallel-Controlled Trial

**DOI:** 10.1155/2016/1064924

**Published:** 2016-06-08

**Authors:** Lei Xiang, Pingping Jiang, Lin Zhou, Xiaomin Sun, Jianlu Bi, Lijuan Cui, Xiaoli Nie, Ren Luo, Xiaoshan Zhao, Yanyan Liu

**Affiliations:** ^1^School of Traditional Chinese Medicine, Southern Medical University, Guangzhou, Guangdong 510515, China; ^2^Department of Traditional Chinese Medicine, Nanfang Hospital, Southern Medical University, Guangzhou, Guangdong 510515, China; ^3^Endocrinology Department, Nanfang Hospital, Southern Medical University, Guangzhou, Guangdong 510515, China; ^4^Second Chinese Medicine Hospital of Guangdong, Guangzhou, Guangdong 510095, China; ^5^First People's Hospital of Baiyun District, Guangzhou, Guangdong 510410, China

## Abstract

Albuminuria is characteristic of early-stage diabetic nephropathy (DN). The conventional treatments with angiotensin receptor blockers (ARB) are unable to prevent the development of albuminuria in normotensive individuals with type 2 diabetes mellitus (T2DM).* Purpose*. The present study aimed to evaluate the effect of ARB combined with a Chinese formula Qidan Dihuang grain (QDDHG) in improving albuminuria and Traditional Chinese Medicine Symptom (TCMS) scores in normotensive individuals with T2DM.* Methods*. Eligible patients were randomized to the treatment group and the control group.* Results*. Compared with baseline (week 0), both treatment and control groups markedly improved the 24-hour albuminuria, total proteinuria (TPU), and urinary albumin to creatinine ratio (A/C) at 4, 8, and 12 weeks. Between treatment and the control group, the levels of albuminuria in the treatment group were significantly lower than in the control group at 8 and 12 weeks (*p* < 0.05). In addition, treatment group markedly decreased the scores of TCMS after treatment.* Conclusion*. This trial suggests that QDDHG combined with ARB administration decreases the levels of albuminuria and the scores for TCMS in normotensive individuals with T2DM.

## 1. Introduction

Diabetic nephropathy (DN) is a major microvascular complication of diabetes. The World Health Organization defines it as a leading cause of dialysis and kidney transplant in developed countries [1]. Albuminuria is characteristic of early-stage DN, which, if not treated, progresses to overt proteinuria and the development of end stage renal disease (ESRD) [2, 3]. Although conventional treatments for DN dual blockade strategies have lowered the risk of albuminuria [4], angiotensin-converting enzyme inhibitors (ACEI) and angiotensin receptor blockers (ARB) are not able to prevent the development of albuminuria in normotensive individuals with type 2 diabetes mellitus (T2DM) [5, 6]. Thus, there is an urgent need to find new effective agents to reduce or delay the progress of microalbuminuria to macroalbuminuria in normotensive individuals with T2DM.

In China, it is well known that traditional Chinese medicine (TCM) can produce remarkable results and some TCM have acquired expert consensus [7] and recommendations for reducing microalbuminuria [8, 9]. Therefore, we systematically reviewed twenty-nine TCM clinical randomized controlled trials for reducing albuminuria using meta-analysis [10] and found that Huang Qi, Danshen, Dihuang, Shanyao, and Gan Cao are the five commonly used herbal medicines. Subsequently, we put these medicines together in certain proportions and named the resulting mixture Qidan Dihuang grain (QDDHG) and observed its effect on reducing albuminuria in combination with ARB in normotensive individuals with T2DM.

With this aim, we designed a randomized, parallel-controlled trial to assess the additive effect of combined ARB and QDDHG on lowering albuminuria levels of DN patients in normotensive individuals with T2DM.

## 2. Methods

### 2.1. Participants

Inpatients were recruited from the Nanfang Hospital attached to the Southern Medical University of Guangdong in China, the Traditional Chinese Medicine Hospital of Guangdong in China, the Second Chinese Medicine Hospital of Guangdong in China, and the First People's Hospital of Baiyun District, Guangzhou, Guangdong in China, between June 2012 and June 2014. All subjects provided written informed consent prior to participation in the study, and the subjects were free to withdraw at any time. The trial protocol was approved by the Ethics Committee of Nanfang Hospital and was registered in the Chinese Clinical Trial Registry (trial registration identifier: ChiCTR-TRC-12002756).

Patients eligible for inclusion in the study were as follows: (1) between the ages of 18 and 80 y, (2) with type 2 diabetes, (3) with a serum HbA1c ≤12.5% and fasting blood glucose (FBG) ≤7.8 mmol/L, or 2 h postprandial blood glucose levels (2 h PBG) <11 mmol/L after diet control, exercise therapy, or taking hypoglycemic medicine for one week, (4) with systolic blood pressure (SBP) ≤140 mmHg and diastolic blood pressure (DBP) ≤90 mmHg after taking ARB to control blood pressure, and (5) with a urinary albumin excretion (UAE) of 30–300 mg/24 h. Patients that were excluded are as follows: (1) with known nondiabetic kidney disease, (2) with a fasting plasma triglyceride level of more than 10 mmol/L, (3) with test drug allergies, (4) with abnormal resting electrocardiogram, (5) with a current blood urea nitrogen (BUN) and serum creatinine (Scr) ratio higher than the normal range, or (6) with other severe complications.

### 2.2. Study Design and Procedure

The study was conducted as a randomized, parallel-controlled trial to examine the effects of microalbuminuria in the control group and treatment group. All participants were randomly assigned to either the treatment or control group in a ratio of 1 : 1 using computer generated random numbers without stratification with background characteristics. The random number list was prepared by an investigator with no clinical involvement in the trial. Finally, 102 patients met the standards and were divided into the control group (ARB) and treatment group (QDDHG plus ARB) in accordance with the above principles.

### 2.3. Herbal Formula

QDDHG is composed of five herb ingredients (see Table 1). The QDDHG formula used in this study was manufactured as a herbal extract powder and the grains were packed in aluminum foil and administered orally at a dose of 7 g, twice a day for 12 weeks. Also, the QDDHG was approved by Guangdong Development and Reform Society and the Test Number was (2009) 431.

### 2.4. Intervention

The participants were randomly placed in either the treatment group or the control group, and the intervention period was 12 weeks. Subjects in the treatment group received packages of QDDHG plus ARB tablets. Subjects in the control group were instructed to take ARB tablets. All patients received ARB at least the minimum recommended dosage and the QDDHG instructions were to drink one bag twice per day (half an hour after breakfast and supper).

### 2.5. Measures

The primary efficacy outcomes were 24 h albuminuria levels (24 h Albu). Secondary efficacy outcomes included total TCM symptom scores (total TCMS scores) and 23 specific TCMS scores according to the Guidelines for Clinical Research of Chinese Medicine (New Drug) [11]. Total TCMS scores were the sum of each specific TCMS score. According to the degree of severity, specific TCMS scores were between 0 and 3 points. TCMS severity was assessed by using a TCMS scores scale and was classified into four grades: 0 points = normal manifestation; 1 point = slight TCMS; 2 points = moderate TCMS; and 3 points = severe TCMS (Table 3 and Figure 2). Further efficacy and safety variables were used: changes in SBP, DBP, FPG, 2 h postprandial blood glucose levels (2 h PBG), serum HbA1c (HbA1c), total proteinuria (TPU), urinary albumin to creatinine ratio (A/C), blood urea nitrogen (BUN), Scr, alanine transaminase (ALT), aspartate transaminase (AST), total cholesterol (TC), triglycerides (TG), high density lipoprotein cholesterol (HDL), and low density lipoprotein cholesterol (LDL).

The assessment of 24 h Albu, total TCMS scores, and each specific TCMS score, SBP, DBP, FBG, 2 h PBG, TPU, and A/C were performed at 0, 4, 8, and 12 weeks. Measurements of ALT, AST, BUN, Scr, HDL, LDL, TC, TG, and HbA1c were taken only at 0 and 12 weeks.

### 2.6. Statistical Analysis

For normally distributed values, the quantitative data were summarized using means ± SD (standard deviations); variables between different groups were compared using a *t*-test for independent samples and within group, a paired *t*-test was used to analyze the results in both control or treated groups before (week 0) and after the treatment (week 12). For nonparametric values, continuous data were presented as medians (interquartile range), whereas categorical data were expressed as a number (*n*) and percentage (%). Variables between different groups were compared using the Mann-Whitney *U* test or chi-square test and within group, a Wilcoxon matched-pairs signed-rank test was used to analyze the results in both control and treated groups before (week 0) and after the treatment (week 12). For all analyses, a two-sided *p* value of <0.05 was considered significant. Statistical analyses were performed using SPSS 13.0.

## 3. Results

### 3.1. Patient Characteristics

**Figure 1:**
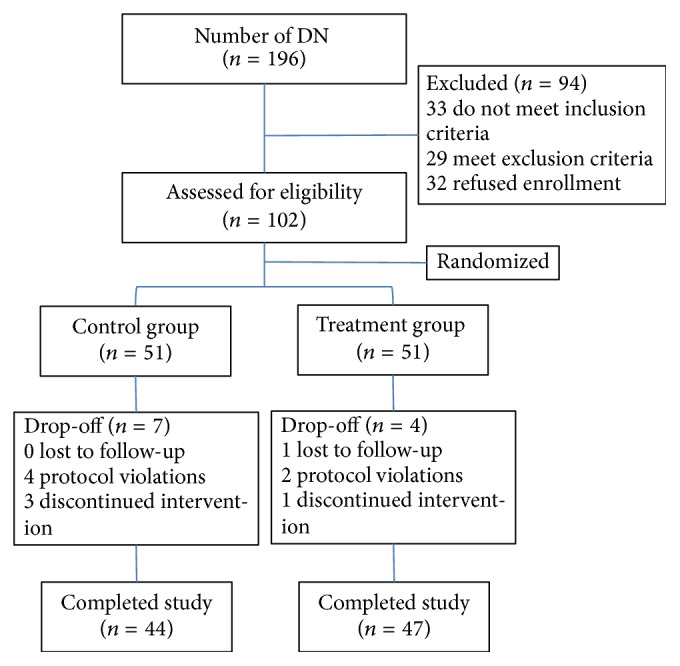
Flow chart of the study population.

The present study included 196 patients who were diagnosed with DN. Ninety-four subjects declined participation or failed to meet inclusion criteria (Figure 1). A total of 102 patients were randomly assigned to the treatment group (*n* = 51) and the control group (*n* = 51). While 91 (89.22%) patients completed the 12-week treatment, one subject (0.98%) was lost to follow-up because of refusal to meet for posttesting. Six subjects (5.88%) violated the protocol due to using other medications. Four subjects (3.92%) discontinued intervention within the two weeks while complaining of no effect after treatment. The two groups did not differ significantly (*p* > 0.05) in any of the baseline characteristics described in Table 2.

### 3.2. Efficacy

#### 3.2.1. Primary Outcome

Baseline variables were not significantly different between the two groups (*p* > 0.05; Table 2). As shown in Table 3, significant reductions in 24 h Albu, TPU, and A/C were recorded in both treatment and control groups after treatment. There was a significant between-group difference in 24 h Albu levels at weeks 8 and 12. The treatment group had significantly reduced 24 h Albu levels over the control group in DN patients (*p* < 0.05, Table 3) and there was no significant difference in FBG, 2 h PBG, TPU, and A/C between the two groups (*p* > 0.05; Table 3).

#### 3.2.2. Secondary Efficacy Outcomes

As shown in Table 4 and Table S1 (see Supplementary Table S1 in the Supplementary Material available online at http://dx.doi.org/10.1155/2016/1064924) (*p* < 0.05), after treatment, treatment group markedly decreased the scores of TCMS in “thirst and need to drink water,” “shortness of breath and disinclination to talk,” “lassitude and lack of strength,” “profuse sweating,” “inability to sleep,” “weakness of waist and knees,” “abdominal distension,” “frequent and excessive urination,” “frequent urination at night,” “uncomfortable with defecation,” and so on, compared with the TCMS scores of baseline (week 0). In contrast, the TCMS scores in the control group were of significant difference in “thirst and need to drink water,” “weakness of waist and knees,” “frequent urination at night,” and so on, compared with the scores of baseline TCMS (week 0).

**Figure 2:**
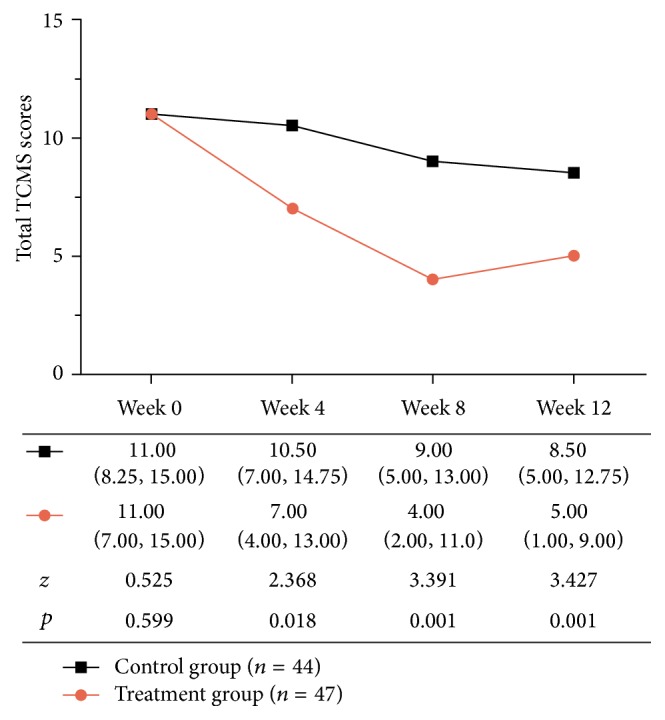
Total Traditional Chinese Medicine Symptom (TCMS) scores during treatment; values are presented as median (interquartile range). *p* values represent the treatment group versus the control group.

The median of total TCMS scores in the treatment group was, respectively, 7.00, 4.00, and 5.00 at 4, 8, and 12 weeks. Compared with the control group, total TCMS scores in the treatment group exhibited a significant difference (*p* < 0.05, Figure 2). Eleven specific TCMS scores out of the total of 23 appeared to be lower in the treatment group than the control group (*p* < 0.05, Table 4). But there was no significant difference between the two groups in the other 12 specific TCMS scores (*p* > 0.05; see Table S1).

#### 3.2.3. Further Efficacy and Safety Variables

Table 5 summarizes other clinical and biochemical characteristics of participants after treatment. Compared with baseline (week 0), both treatment and control groups markedly decreased the SBP, DBP, HbA1c, LDL, TG, and TC at 12 weeks (*p* < 0.05; Table 5). No other significant differences were observed both within the group and between the groups in Table 5 (*p* > 0.05).

### 3.3. Adverse Events

Adverse events occurred in 1 subject in the treatment group and in 3 subjects in the control group, leading to study discontinuation. In the treatment group, 1 subject had insomnia. In the control group, 1 case of diarrhea and 2 cases of dizziness occurred. Finally, no other drug-related serious adverse events occurred in this study.

## 4. Discussion

### 4.1. Principal Findings

In this randomized trial of QDDHG and ARB for early-stage DN in normotensive individuals with T2DM continuing to experience albuminuria, we noted statistically significant benefits associated with interventions. The levels of albuminuria in the treatment group were significantly lower than in the control group at 8 and 12 weeks. Furthermore, significant benefits of total TCMS scores were observed in the treatment group. Eleven specific TCMS scores were significantly lower than in the control group after intervention. The change of scores indicated the symptoms of patients significantly better than in the control group according to TCM theory. Moreover, the results showed ALT, AST, BUN, and Scr to be within the normal range. No serious adverse events related to this study were reported.

### 4.2. Relationship to the Literature

In a recent review of diabetic kidney disease, studies show that approximately 80–90% of patients with albuminuria progress to more advanced stages [12]. The degree of reduction in albuminuria is correlated with a decline of glomerular filtration rate (GFR) [13]. In outcome trials of patients with diabetic nephropathy, retrospective analyses demonstrate a robust relationship between the magnitude of albuminuria reduction [14, 15] and the slowing of chronic kidney diseases (CKD) progression as well as reduced cardiovascular event rates [16–19]. ARB could reduce the levels of albuminuria but not be able to prevent the development of microalbuminuria in normotensive individuals with T2DM and T1DM [5, 6]. It may be possible that ARB improve proteinuria by lowering blood pressure. So, within the normal scope of blood pressure, the change of albuminuria was limited. These results were consistent with our control group. When combining QDDHG with ARB intervention, the treatment group had a lower albuminuria level at an earlier date. This may provide a new treatment method for albuminuria in normotensive individuals with T2DM.

It is well known that TCM has been used for thousands of years for kidney disease in China. Examples include* Astragalus membranaceus*; a meta-analysis that comprised 21 randomized controlled trails and 4 controlled clinical trials included 1804 patients (945 in the treatment group and 859 in the control group) and showed a greater therapeutic effect in reducing the serum albuminuria level of DN patients [20]. QDDHG was a new formulation and composed of Huang Qi, Danshen, Dihuang, Shanyao, and Gan Cao. The five types of traditional medicine come from the evidence-based research that are widely used to treat proteinuria [10]. Dihuang is sovereign medicinal, playing the role of clearing heat, cooling the blood, nourishing yin, and engendering fluid. Both Huang Qi and Danshen are minister medicinal. Huang Qi can tonify qi, secure the exterior, and induce diuresis to alleviate edema. Danshen can activate blood and dissipate stasis. Shanyao is assistant medicinal and can fortify the spleen and nourish yin. Gan Cao is courier medicinal and can tonify qi and harmonize the middle. The collaborative role of the five medicines together is tonifying qi, nourishing yin, and activating blood. These functions are consistent with the treatment principles of TCM. In addition, animal studies have also shown that Danshen [21], Dihuang [22], and Glycyrrhiza [23] and its active components are effective in reducing albuminuria levels and ameliorating the pathological changes of early DN in rat models. The current study showed that the effects of QDDHG in reducing albuminuria were similar to those in the studies mentioned above.

Furthermore, we found that, in the treatment group, the TCMS were remarkably improved with regard to scores, such as thirst and need to drink water, shortness of breath and disinclination to talk, lassitude and lack of strength, profuse sweating, inability to sleep, the weakness of waist and knees, abdominal distension, frequent and excessive urination, frequent urination at night, discomfort with defecation, and frequent and excessive number of stools, and were significantly less than the control group. The TCMS scores were used to judge variation in patient symptoms. The higher the score in certain symptoms, the more severe the degree of the disease. In this study, the scores of the treatment group were less than the control group in 11 items. These results illustrate that TCM symptoms were very obviously improved in this trial. Of course the other 12 items showed no clear difference between the two groups. It may be that the QDDHG primarily reduced albuminuria levels and did not affect other symptoms in this study.

### 4.3. Strengths and Limitations

The present study was a randomized clinical trial and was directed against albuminuria of DN in normotensive individuals with T2DM. All participants' systolic and diastolic blood pressure was ≤140/90 mmHg and the levels of albuminuria were 30–300 mg/24 h. For this early-stage DN, we focused on observing the effect of different treatments for preventing the progress from microalbuminuria to macroalbuminuria. On the basis of conventional ARB therapy, we added QDDHG to the treatment group. QDDHG was composed of the five types of traditional medicine that are widely used to treat proteinuria. Furthermore, we used the TCMS scale to evaluate the therapeutic effects of intervention.

Our study had certain limitations. First, it did not recruit a sufficient number of participants that met with inclusion criteria. The number of participants was small. At the same time, the short-term intervention of this study did not allow the formation of definite conclusions on the long-term effects of different treatments on DN progression. Moreover, the mechanisms underlying the efficacy of QDDHG are yet to be clarified.

### 4.4. Implications for Clinical Practice and Future Research

Our study demonstrated that QDDHG has an additive effect on reducing albuminuria levels and improving TCMS scores. It could be combined with ARB to treat early-stage DN in normotensive individuals with T2DM or T1DM. In further research, we need to follow up these participants and observe the long-term effects between the two groups. Moreover, the underlying mechanisms of QDDHG need to be clarified.

## 5. Conclusions

In summary, this trial suggests that QDDHG combined with ARB decreases the levels of albuminuria and TCMS scores in normotensive individuals with T2DM.

## Supplementary Material

The other 12 items showed no clear difference between the treatment and the control group in Traditional Chinese Medicine Symptom score.

## Figures and Tables

**Table 1:** Nomenclature of the Chinese herbs in Qidan Dihuang grain (QDDHG).

Chinese pinyin	Pharmaceutical name	Latin botanical name	Proportion (%)
Huang Qi	Membranous milkvetch root	Radix Astragali	37.5
Danshen	Danshen root	Radix Salviae Miltiorrhizae	18.75
Dihuang	Rehmannia root	Radix Rehmanniae	18.75
Shanyao	Common Yam Rhizome	Rhizoma Diosscoreae	18.75
Gan Cao	Liquorice root	Radix Glycyrrhizae	6.25

**Table 2:** Patient baseline characteristics.

Characteristic	Treatment group (*n* = 47)	Control group (*n* = 44)	*p* value
Age (y)	57.21 ± 13.20	58.16 ± 11.59	0.742
Male sex, no. (%)	24 (51.1)	22 (50)	0.919
BMI (kg/m^2^)	25.27 ± 2.88	24.70 ± 2.85	0.342
Course of DN (y)	0.10 (0.10, 1.30)	0.20 (0.10, 0.70)	0.627
SBP (mmHg)	131.00 (124.00, 136.00)	133.50 (120.00, 138.00)	0.518
DBP (mmHg)	80.00 (75.00, 85.00)	80.00 (73.00, 85.00)	0.378
Heart rate (bpm)	80.00 (76.00, 85.00)	78.00 (74.25, 82.00)	0.115
Albu (mg/24 h)	85.30 (66.00, 176.30)	90.50 (67.78, 124.68)	0.940
TPU (g/24 h)	0.20 (0.10, 0.30)	0.20 (0.20, 0.30)	0.943
A/C (mg/mol)	20.70 (11.00, 30.50)	19.45 (7.83, 30.63)	0.952
FPG (mmol/L)	7.40 (6.70, 7.80)	7.40 (6.80, 7.80)	0.883
2 h PBG (mmol/L)	9.52 ± 1.27	9.58 ± 1.05	0.877
HbA1c (%)	8.92 ± 1.74	8.78 ± 2.09	0.717
TG (mmol/L)	1.43 (1.15, 2.30)	1.55 (0.95, 2.29)	0.799
TC (mmol/L)	5.06 ± 1.09	5.11 ± 1.02	0.828
LDL (mmol/L)	3.11 ± 0.86	3.06 ± 1.03	0.798
HDL (mmol/L)	1.20 (0.99, 1.49)	1.12 (0.92, 1.64)	0.700
BUN (mmol/L)	5.46 ± 1.86	5.28 ± 1.49	0.605
Scr (*μ*mol/L)	63.00 (56.00, 80.00)	62.00 (55.00, 87.25)	0.694
ALT (*μ*/L)	18.00 (14.00, 27.00)	19.50 (15.00, 25.75)	0.889
AST (*μ*/L)	20.00 (15.00, 27.00)	19.5 (16.25, 24.00)	0.769

Data are expressed as the mean ± SD for normal distribution. Nonnormally distributed values are presented as medians (interquartile range). *p* values represent the treatment group versus the control group. BMI, body mass index; DN, diabetic nephropathy; SBP, systolic blood pressure; DBP, diastolic blood pressure; Albu, albuminuria; TPU, total proteinuria; A/C, urinary albumin to creatinine ratio; FPG, fasting plasma glucose; PBG, postprandial blood glucose; TG, triglycerides; TC, total cholesterol; LDL, low density lipoprotein cholesterol; HDL, high density lipoprotein cholesterol; BUN, blood urea nitrogen; Scr, serum creatinine; ALT, alanine transaminase; AST, aspartate transaminase.

**Table 3:** Change in 24-hour albuminuria levels and other relative indicators.

	Week	Treatment group	Control group	*p* value
		(*n* = 47)	(*n* = 44)	
Albu (mg/24 h)	0	85.30 (66.00, 176.30)	90.50 (67.78, 124.68)	0.940
	4	61.50 (49.00, 110.20)^*∗*^	89.00 (61.78, 125.25)	0.086
	8	51.00 (37.00, 90.00)^*∗*^	70.00 (53.00, 100.93)^*∗*^	0.031
	12	41.40 (29.00, 68.00)^*∗*^	47.65 (36.30, 100.53)^*∗*^	0.045

FBG (mmol/L)	0	7.40 (6.70, 7.80)	7.40 (6.80, 7.80)	0.883
	4	6.80 (6.10, 7.50)	7.00 (6.15, 7.60)	0.343
	8	6.90 (6.00, 7.40)	6.80 (5.85, 7.60)	0.899
	12	7.00 (6.30, 7.6)	6.75 (6.10, 7.53)	0.775

2 h PBG (mmol/L)	0	9.52 ± 1.27	9.58 ± 1.05	0.877
	4	9.48 ± 0.28	9.49 ± 0.25	0.988
	8	9.37 ± 0.27	9.45 ± 0.26	0.836
	12	9.28 ± 0.29	9.42 ± 0.34	0.952

TPU (g/24 h)	0	0.20 (0.10, 0.30)	0.20 (0.20, 0.30)	0.943
	4	0.10 (0.10, 0.20)^*∗*^	0.10 (0.10, 0.30)^*∗*^	0.792
	8	0.10 (0.10, 0.20)^*∗*^	0.10 (0.10, 0.20)^*∗*^	0.936
	12	0.10 (0.10, 0.20)^*∗*^	0.10 (0.10, 0.20)^*∗*^	0.633

A/C (mg/mol)	0	20.70 (11.00, 30.50)	19.45 (7.83, 30.63)	0.952
	4	16.30 (8.10, 25.00)^*∗*^	16.10 (6.05, 23.45)^*∗*^	0.724
	8	15.00 (7.20, 20.60)^*∗*^	11.15 (5.40, 20.80)^*∗*^	0.380
	12	10.10 (5.60, 17.00)^*∗*^	8.9 (5.30, 19.23)^*∗*^	0.662

Data are expressed as the mean ± SD for normal distribution. Nonnormally distributed values are presented as median (interquartile range). *p* values represent the treatment group versus the control group. *∗* represent *p* < 0.05 versus baseline (week 0), paired *t*-test for normal distribution or Wilcoxon matched-pairs signed-rank test for nonnormal distribution. Albu, albuminuria; FPG, fasting plasma glucose; PBG, postprandial blood glucose; TPU, total proteinuria; A/C, urinary albumin to creatinine ratio.

**Table 4:** Significant differences in 11 specific Traditional Chinese Medicine Symptom (TCMS) scores.

TCMS	Week	Treatment group	Control group	*z*	*p* value
		(*n* = 47)	(*n* = 44)		
Thirst and need to drink water	0	1 (1.00, 2.00)	1 (1.00, 1.75)	0.289	0.773
	4	0 (0.00, 2.00)^*∗*^	1 (1.00, 1.00)^*∗*^	1.639	0.101
	8	0 (0.00, 1.00)^*∗*^	1 (0.00, 1.00)^*∗*^	2.757	0.006
	12	0 (0.00, 1.00)^*∗*^	1 (0.00, 1.00)^*∗*^	2.786	0.005

Shortness of breath and disinclination to talk	0	0 (0.00, 1.00)	1 (0.00, 1.00)	1.266	0.205
	4	0 (0.00, 1.00)^*∗*^	1 (0.00, 1.00)	2.586	0.010
	8	0 (0.00, 0.00)^*∗*^	0 (0.00, 1.00)	2.204	0.028
	12	0 (0.00, 0.00)^*∗*^	0 (0.00, 1.00)	2.164	0.030

Lassitude and lack of strength	0	1 (0.00, 1.00)	1 (0.00, 1.00)	0.498	0.618
	4	1 (0.00, 1.00)^*∗*^	1 (0.25, 1.00)	1.675	0.094
	8	0 (0.00, 1.00)^*∗*^	1 (0.00, 1.00)	1.874	0.061
	12	0 (0.00, 1.00)^*∗*^	1 (0.00, 1.00)	2.347	0.019

Profuse sweating	0	1 (0.00, 1.00)	1 (0.00, 1.00)	0.014	0.989
	4	0 (0.00, 1.00)^*∗*^	1 (0.00, 1.00)	2.944	0.003
	8	0 (0.00, 1.00)^*∗*^	1 (0.00, 1.00)	3.377	0.001
	12	0 (0.00, 0.00)^*∗*^	1 (0.00, 1.00)	4.060	0.000

Inability to sleep	0	1 (0.00, 1.00)	1 (0.00, 1.00)	0.237	0.812
	4	0 (0.00, 1.00)^*∗*^	1 (0.00, 1.00)	1.555	0.120
	8	0 (0.00, 1.00)^*∗*^	1 (0.00, 1.00)	2.081	0.037
	12	0 (0.00, 1.00)^*∗*^	1 (0.00, 1.00)	2.188	0.029

Weakness of waist and knees	0	1 (1.00, 1.00)	1 (0.25, 1.00)	0.233	0.816
	4	0 (0.00, 1.00)^*∗*^	1 (0.00, 1.00)	1.716	0.086
	8	0 (0.00, 1.00)^*∗*^	1 (0.00, 1.00)^*∗*^	2.057	0.040
	12	0 (0.00, 1.00)^*∗*^	1 (0.00, 1.00)^*∗*^	2.647	0.008

Abdominal distension	0	0 (0.00, 0.00)	0 (0.00, 0.75)	1.112	0.911
	4	0 (0.00, 0.00)	0 (0.00, 1.00)	2.403	0.016
	8	0 (0.00, 0.00)^*∗*^	0 (0.00, 1.00)	2.598	0.009
	12	0 (0.00, 0.00)	0 (0.00, 1.00)	2.042	0.041

Frequent and excessive urination	0	1 (0.00, 1.00)	1 (0.00, 1.00)	0.621	0.534
	4	0 (0.00, 1.00)	1 (1.00, 1.00)	1.673	0.094
	8	0 (0.00, 1.00)^*∗*^	1 (0.25, 1.00)	2.513	0.012
	12	0 (0.00, 1.00)^*∗*^	1 (0.00, 1.00)	2.607	0.009

Frequent urination at night	0	1 (0.00, 2.00)	1 (1.00, 1.00)	0.189	0.850
	4	1 (0.00, 1.00)^*∗*^	1 (1.00, 1.00)^*∗*^	1.145	0.252
	8	0 (0.00, 1.00)^*∗*^	1 (0.00, 1.00)^*∗*^	2.085	0.037
	12	0 (0.00, 1.00)^*∗*^	1 (0.00, 1.00)^*∗*^	2.115	0.034

Uncomfortable with defecation	0	0 (0.00, 0.00)	0 (0.00, 0.00)	0.476	0.634
	4	0 (0.00, 0.00)	0 (0.00, 1.00)	1.975	0.048
	8	0 (0.00, 0.00)	0 (0.00, 1.00)	3.114	0.002
	12	0 (0.00, 0.00)^*∗*^	0 (0.00, 1.00)	2.984	0.003

Frequent and excessive number of stools	0	0 (0.00, 0.00)	0 (0.00, 0.00)	1.116	0.264
	4	0 (0.00, 0.00)	0 (0.00, 0.00)	1.972	0.049
	8	0 (0.00, 0.00)	0 (0.00, 0.00)	2.214	0.027
	12	0 (0.00, 0.00)	0 (0.00, 0.75)	2.374	0.018

Data are expressed as median (interquartile range). *p* values represent the treatment group versus the control group. *∗* represent *p* < 0.05 versus baseline (week 0), Wilcoxon matched-pairs signed-rank test for nonnormal distribution.

**Table 5:** The results of the two groups on the further efficacy and safety variables.

	Treatment group	Control group	*p* value
	(*n* = 47)	(*n* = 44)	
SBP (mmHg)	119.00 (115.00, 127.00)^*∗*^	119.00 (109.25, 127.00)^*∗*^	0.415
DBP (mmHg)	78.00 (75.00, 80.00)^*∗*^	76.00 (72.00, 80.00)^*∗*^	0.111
HbA1c (%)	7.67 ± 0.24^*∗*^	7.50 ± 0.20^*∗*^	0.066
AST (*μ*/L)	20.00 (16.00, 25.00)	18.50 (16.00, 23.75)	0.650
ALT (*μ*/L)	18.0 (14.00, 26.00)	19.50 (15.25, 24.50)	0.827
BUN (mmol/L)	5.19 ± 0.18	5.13 ± 0.24	0.832
Scr (*μ*mol/L)	63.00 (56.00, 75.00)	62.00 (54.25, 80.75)	0.921
HDL (mmol/L)	1.23 (1.11, 1.46)	1.20 (0.99, 1.65)	0.586
LDL (mmol/L)	2.58 ± 0.10^*∗*^	2.77 ± 0.16^*∗*^	0.296
TG (mmol/L)	1.22 (0.87, 1.56)^*∗*^	1.14 (0.53, 1.80)^*∗*^	0.984
TC (mmol/L)	4.44 ± 0.13^*∗*^	4.67 ± 0.17^*∗*^	0.136

Data are expressed as the mean ± SD for normal distribution. Nonnormally distributed values are presented as median (interquartile range). *p* values represent the treatment group versus the control group. *∗* represent *p* < 0.05 versus baseline (week 0), paired *t*-test for normal distribution or Wilcoxon matched-pairs signed-rank test for nonnormal distribution. SBP, systolic blood pressure; DBP, diastolic blood pressure; AST, aspartate transaminase. ALT, alanine transaminase; BUN, blood urea nitrogen; Scr, serum creatinine; HDL, high density lipoprotein cholesterol; LDL, low density lipoprotein cholesterol; TG, triglycerides; TC, total cholesterol.
