# Supplementary material for: Additive Effect of Qidan Dihuang Grain, a Traditional Chinese Medicine, and Angiotensin Receptor Blockers on Albuminuria Levels in Patients with Diabetic Nephropathy: A Randomized, Parallel-Controlled Trial
Source: Evid Based Complement Alternat Med. 2016 Jun 8;2016:1064924. doi: 10.1155/2016/1064924 (PMC4916306; doi:10.1155/2016/1064924)
Supplement: Supplementary file 1 — The other 12 items showed no clear difference between the treatment and the control group in Traditional Chinese Medicine Symptom score. [file 1064924.f1.docx]

**Table S1. Twelve symptoms showing no significant difference in Traditional Chinese Medicine Symptom score**

| TCMS | Week | Treatment group (n = 47) | Control group (n = 44) | z | *p* -value |
| --- | --- | --- | --- | --- | --- |
| Dry stool | 0 | 0 (0.00, 1.00) | 0 (0.00, 1.00) | 1.444 | 0.149 |
|  | 4 | 0 (0.00, 0.00) | 0 (0.00, 1.00) | 1.819 | 0.069 |
|  | 8 | 0 (0.00, 0.00) | 0 (0.00, 0.00)* | 0.895 | 0.371 |
|  | 12 | 0 (0.00, 0.00) | 0 (0.00, 0.00)* | 1.057 | 0.291 |
| Numbness of limbs and trunk | 0 | 1 (0.00, 1.00) | 1 (0.00, 1.00) | 0.715 | 0.474 |
|  | 4 | 0 (0.00, 1.00) | 0.5 (0.00, 1.00) | 0.966 | 0.334 |
|  | 8 | 0 (0.00, 1.00)* | 0 (0.00, 1.00)* | 1.082 | 0.279 |
|  | 12 | 0 (0.00, 1.00)* | 0 (0.00, 1.00) | 0.199 | 0.842 |
| Swift digestion with rapid return to hunger | 0 | 0 (0.00, 1.00) | 0 (0.00, 1.00) | 1.729 | 0.084 |
|  | 4 | 0 (0.00, 0.00)* | 0 (0.00, 0.75)* | 0.767 | 0.443 |
|  | 8 | 0 (0.00, 0.00)* | 0 (0.00, 0.00)* | 1.351 | 0.177 |
|  | 12 | 0 (0.00, 0.00)* | 0 (0.00, 0.00)* | 0.178 | 0.859 |
| Heat in the palms and soles of stool | 0 | 0 (0.00, 0.00) | 0 (0.00, 1.00) | 1.055 | 0.291 |
|  | 4 | 0 (0.00, 0.00) | 0 (0.00, 0.00)* | 0.749 | 0.454 |
|  | 8 | 0 (0.00, 0.00)* | 0 (0.00, 0.00)* | 1.174 | 0.240 |
|  | 12 | 0 (0.00, 0.00)* | 0 (0.00, 0.00)* | 1.297 | 0.195 |
| Forgetfulness | 0 | 0 (0.00, 1.00) | 0 (0.00, 1.00) | 1.608 | 0.108 |
|  | 4 | 0 (0.00, 1.00) | 0 (0.00, 1.00) | 1.356 | 0.175 |
|  | 8 | 0 (0.00, 0.00) | 0 (0.00, 1.00) | 1.544 | 0.123 |
|  | 12 | 0 (0.00, 0.00) | 0 (0.00, 1.00) | 1.615 | 0.106 |
| Edema | 0 | 0 (0.00, 0.00) | 0 (0.00, 0.00) | 0.071 | 0.944 |
|  | 4 | 0 (0.00, 0.00) | 0 (0.00, 0.00) | 0.090 | 0.928 |
|  | 8 | 0 (0.00, 0.00) | 0 (0.00, 0.00) | 0.155 | 0.877 |
|  | 12 | 0 (0.00, 0.00) | 0 (0.00, 0.00) | 0.122 | 0.903 |
| Heavy headedness and heavy body | 0 | 0 (0.00, 1.00) | 0 (0.00, 1.00) | 0.304 | 0.761 |
|  | 4 | 0 (0.00, 0.00) | 0 (0.00, 1.00) | 1.578 | 1.114 |
|  | 8 | 0 (0.00, 0.00) | 0 (0.00, 1.00) | 1.624 | 0.104 |
|  | 12 | 0 (0.00, 0.00)* | 0 (0.00, 1.00) | 1.446 | 0.148 |
| Palpitation | 0 | 0 (0.00, 0.00) | 0 (0.00, 1.00) | 1.246 | 0.213 |
|  | 4 | 0 (0.00, 0.00) | 0 (0.00, 0.75) | 1.138 | 0.255 |
|  | 8 | 0 (0.00, 0.00) | 0 (0.00, 0.00) | 0.953 | 0.341 |
|  | 12 | 0 (0.00, 0.00) | 0 (0.00, 0.00)* | 0.420 | 0.674 |
| Vexation | 0 | 0 (0.00, 1.00) | 0 (0.00, 1.00) | 0.711 | 0.477 |
|  | 4 | 0 (0.00, 1.00) | 1 (0.00, 1.00) | 0.778 | 0.436 |
|  | 8 | 0 (0.00, 0.00) | 0 (0.00, 0.00) | 0.258 | 0.796 |
|  | 12 | 0 (0.00, 0.00)* | 0 (0.00, 0.75) | 1.203 | 0.229 |
| Lumbago and back pain | 0 | 0 (0.00, 1.00) | 1 (0.00, 1.00) | 1.717 | 0.086 |
|  | 4 | 0 (0.00, 1.00) | 0 (0.00, 1.00) | 1.235 | 0.217 |
|  | 8 | 0 (0.00, 0.00) | 0 (0.00, 1.00) | 1.733 | 0.083 |
|  | 12 | 0 (0.00, 1.00) | 0 (0.00, 1.00)* | 0.450 | 0.652 |
| Chest pain and hypochondriac pain | 0 | 0 (0.00, 0.00) | 0 (0.00, 0.00) | 1.392 | 0.164 |
|  | 4 | 0 (0.00, 0.00) | 0 (0.00, 0.00) | 1.716 | 0.086 |
|  | 8 | 0 (0.00, 0.00) | 0 (0.00, 0.00) | 1.085 | 0.278 |
|  | 12 | 0 (0.00, 0.00) | 0 (0.00, 0.00) | 1.449 | 0.147 |
| General body ache | 0 | 0 (0.00, 1.00) | 0 (0.00, 0.75) | 1.536 | 0.125 |
|  | 4 | 0 (0.00, 1.00) | 0 (0.00, 1.00) | 1.012 | 0.311 |
|  | 8 | 0 (0.00, 1.00)* | 0 (0.00, 1.00) | 0.431 | 0.667 |
|  | 12 | 0 (0.00, 1.00)* | 0 (0.00, 1.00) | 0.190 | 0.849 |

Data are expressed as median (interquartile range). *p*-values represent the treatment group vs. the control group. * represent p <0.05 vs. baseline (week 0), Wilcoxon matched-pairs signed-rank test for non-normally distribution.
